# Supplementary material for: Emergence and control of photonic band structure in stacked OLED microcavities
Source: Nat Commun. 2021 Oct 20;12:6111. doi: 10.1038/s41467-021-26440-3 (PMC8528838; doi:10.1038/s41467-021-26440-3)
Supplement: Supplementary file 4 — Supplementary Data 1 [file 41467_2021_26440_MOESM4_ESM.zip › OLED Simulation v2-1/OLED Simulation/Materials Data/Materials Database/rii-database-2019-02-11.zip!/database/info/main/HgS.html]

# Mercury sulfide, HgS

## Other names

- Mercuric sulfide
- Mercury sulphide
- Mercury(II) sulfide
- Cinnabar, Vermilion

## Mineral

- Cinnabar

## External links

- Mercury sulfide - Wikipedia
- Mercury sulphide - WebElements
- Cinnabar - Wikipedia
- Vermilon - Wikipedia
